# Long-term graft function following autologous hematopoietic cell transplantation and the impact of preemptive plerixafor in predicted poor mobilizers

**DOI:** 10.1038/s41408-018-0050-2

**Published:** 2018-01-29

**Authors:** Alissa Visram, Christopher Bredeson, David Allan, Mitchell Sabloff, Lothar Huebsch, Jason Tay, Natasha Kekre, Sheryl McDiarmid, Ranjeeta Mallick, Alan Tinmouth, Lisa Martin, Linda Hamelin, Dawn Maze

**Affiliations:** 1 Department of Hematology, The Ottawa Hospital, Ottawa, Canada; 2 Department of Medicine, Calgary, Canada; 3 Ottawa Hospital Research Institute, Ottawa, Canada; 4 School of Epidemiology, Public Health and Preventive Medicine, University of Ottawa, Ottawa, Canada; 5 Canadian Blood Services Stem Cell Processing Laboratory, Ottawa, Canada

It is generally accepted that peripheral blood autologous hematopoietic cell transplantation (AHCT) requires a minimum of 2 × 10^6^ CD34+ cells/kg for successful engraftment in the early post-transplant period^1–3^. The American Society for Blood and Marrow Transplant (ASBMT) recommends a target dose of 3–5 × 10^6^ CD34+ cells/kg^1^. Prior studies have shown that infusion of fewer CD34+ cells results in poor hematopoietic function at 6 and 12 months^4, 5^; however, whether there is an optimal CD34+ dose needed to sustain long-term graft function has not been established.

This study sought to establish the minimum number of CD34+ cells/kg required for, and to identify factors that may be predictive of, long-term hematopoietic function. A secondary objective was to assess the long-term outcomes of AHCT following preemptive use of plerixafor.

A retrospective review of all autologous collections between January 2004 and September 2013 at The Ottawa Hospital was performed. All patients included in the study had consented to having their data collected. Patients were excluded if they did not proceed to AHCT, were not followed locally, or if the AHCT was for a non-hematological indication. The study was approved by the institution’s Research Ethics Board. Blood counts were collected at 6, 12, 24, 36, 48, and 60 months (with a 30-day margin of error if >6 months post-transplant) after the date of AHCT and until either relapse or January 2016 (the study end date). Poor long-term hematopoietic function was defined as an absolute neutrophil count (ANC) <1 × 10^9^/L, hemoglobin <100 g/L, or platelets <100 × 10^9^/L.

After May 2009, plerixafor became available through a special access program. Though there was variation between physician practices, the general institutional practice was to use plerixafor preemptively for patients with a pre-collection CD34+ count of <2 × 10^6^ cells/kg, i.e., predicted poor mobilizers (PPMs)^6^. The pre-collection CD34+ count was determined by dividing the number of CD34+ cells/µL by the patient’s weight to predict the number of CD34+ cells obtained with 10-L apheresis. To study the impact of preemptive plerixafor, clinical outcomes of PPMs who received plerixafor were compared to those of PPMs prior to plerixafor availability. The collection procedure was performed as previously described^7^. Data on post-transplant transfusion requirements, culture-positive infections, and infections requiring hospital admission were collected.

Logistic regression was used to analyze the factors associated with poor long-term graft function. Chi-square tests were used to analyze the number of patients with poor long-term graft function at 1, 2, 3, 4, and 5 years based on infused CD34+ cell dose and to assess the differences in clinical outcomes between PPM and plerixafor-mobilized patients. The median CD34+ dose, CD34+ cell yield, and peripheral blood counts of these groups were compared using the Mann–Whitney rank sum test.

The study included 560 patients (Table 1). The median pre-collection CD34+ count was 3.12 × 10^6^ (range 0–63.11) cells/kg and the median CD34+ dose collected was 6.41 × 10^6^ (range 0.31–58.77) cells/kg. The median follow up was 24 (range 0.7–63) months. In total, 297 (53%) patients relapsed during the study period. At 1 and 5 years post AHCT, 357 and 96 patients were included, respectively.

**Table 1:** Baseline characteristics of all study patients

	Multiple myeloma (*N* = 210)	Lymphoma (*N* = 350)	All patients (*N* = 560)
*Disease (%)*			
Multiple myeloma	210 (100)	Na	210 (37.5)
Indolent NHL	Na	92 (26.3)	92 (16.4)
Aggressive NHL	Na	197 (56.3)	197 (35.2)
Hodgkin’s lymphoma	Na	61 (17.4)	61 (10.9)
*Median age at collection—years (range)*	58 (31–69)	52 (14–71)	55 (14–71)
*Gender —no.* *(%)*			
Male	131 (62.4)	209 (59.7)	340 (60.7)
Female	79 (37.6)	141 (40.3)	220 (39.3)
*Disease status at mobilization—no.* *(%)*			
CR	9 (4.3)	53 (15.1)	62 (11)
PR	62 (29.5)	75 (21.4)	137 (24.5)
Chemosensitive	17 (8.1)	22 (6.3)	39 (7)
Rel-ref	4 (1.9)	139 (39.7)	143 (25.5)
Unknown	118 (56.2)	61 (17.4)	179 (32)
*No. chemotherapy lines prior to collection (%)*			
1	185 (88.1)	168 (48)	353 (63)
2	9 (4.3)	140 (40)	149 (26.6)
≥3	2 (1)	38 (10.6)	40 (7.1)
Unknown	14 (6.7)	4 (1.1)	18 (3.2)
*Mobilization regimen—no.* *(%)*			
Cyclo-G	206 (98.1)	165 (47.1)	371 (66.3)
DHAP-G	0(0)	102 (29.1)	102 (18.2)
ICE-G	0(0)	32 (9.1)	32 (5.7)
Other chemotherapy-GSF	0(0)	18 (5.1)	18 (3.2)
Plerixafor	2 (1)	23 (6.6)	25 (4.5)
Other	2 (1)	10 (2.9)	12 (2.1)
*No. prior collections* *(%)*			
0	203 (96.7)	336 (96)	539 (96.3)
1	6 (2.9)	14 (4)	20 (3.6)
2	1 (0.5)	0(0)	1 (0.1)
*Median CD34+ counts—×10* ^*6*^ *cells/kg (range)*			
Pre-collection	4.02 (0.29–38.97)	2.61 (0–63.11)	3.12 (0–63.11)
Total collected	8.15 (1.05–32.67)	5.59 (0.32–58.77)	6.41 (0.32–58.77)

*Cyclo-G* cyclophosphamide and G-CSF, *DHAP-G* dexamethasone, cytarabine, cisplatinum, G-GCSF, *ICE-G* ifosfamide, carboplatin, etoposide, G-CSF, *CR* complete remission, *PR* partial remission, *Rel-ref* relapse refractory

The percent of patients who had poor hematopoietic function at 1, 3, and 5 years was 13.4% (*n* = 48), 7.2% (*n* = 13), and 9.4% (*n* = 9), respectively. At 1 year post-transplant, the proportion of patients with poor hematopoietic function was significantly higher in patients who received fewer than 3 × 10^6^ CD34+ cells/kg (24.4%) compared to patients who received 5–10 × 10^6^ CD34+ cells/kg (11%, *p* = 0.028) or >10 × 10^6^ CD34+ cells/kg (6.5%, *p* = 0.019, Table 2). Though patients who received lower CD34+ doses initially had poorer graft function, beyond 1 year post-transplant, there was no statistically significant difference in hematopoietic function based on the number of CD34+ cells infused. There was no significant difference in the relapse rates based on quantity of CD34+ cells infused.

**Table 2:** Long-term hematopoietic outcomes of all non-relapsed study patients

	Years post HSCT				
	**1** (*N* = 357)	**2** (*N* = 280)	**3** (*N* = 180)	**4** (*N* = 131)	**5** (*N* = 96)
Median hemoglobin—g/L (range)	129 (10–163)	130 (76–168)	134 (79–168)	132 (79–166)	133 (91–171)
Median platelets—<100 × 10^9^/L (range)	179 (21–449)	178 (32–457)	193 (16–468)	185 (42–420)	173 (48–446)
Median ANC—×10^9^/L (range)	3.3 (0.17–10.5)	3.4 (1–22.6)	3.4 (1.2–11.1)	3.1 (1.3–11.2)	3.3 (0.35–19.2)
Poor hematopoietic function*—total no. *(%)*	48 (13.4)	31 (11)	13 (7.2)	10 (7.6)	9 (9.4)
Thrombocytopenia—no.	27	16	7	7	4
Anemia—no.	9	11	5	2	3
Neutropenia—no.	2	0	0	0	1
>1 cytopenia—no.	10	4	1	1	1
Poor hematopoietic function* based on CD34+ infusion dose (in ×10 ^*6*^ *cells/kg)*					
0–2.99—no. (%)	10/41 (24.4)	6/33 (18.2)	1/20 (5)	3/16 (18.8)	3/13 (23.1)
3–4.99—no. (%)	18/116 (15.5)	9/87 (10.3)	5/59 (8.5)	3/47 (6.4)	3/30 (10)
5–9.99—no. (%)	17/154 (11)	14/119 (11.8)	6/76 (7.9)	3/50 (6)	3/35 (8.6)
≥10—no. (%)	3/46 (6.5)	2/41 (4.9)	1/25 (4)	1/18 (5.5)	0/18(0)

*HSCT* hematopoietic stem cell transplantation

^*^Poor hematopoietic function was defined as neutropenia (ANC <1 × 10^9^/L), anemia (hemoglobin <100 g/L), or thrombocytopenia (platelets <100 × 10^9^/L)

^**^The percent of patients with poor hematopoietic function was determined by stratifying patients into categories based on the CD34 dose they were given, and then dividing the number of patients who met the criteria for poor hematopoietic function at each time point by the total number of patients included in the study at that time point who received the same CD34 dose

Ten patients received fewer than 2 × 10^6^ CD34+ cells/kg. Of these, 4 patients died within 1 month of AHCT (1 from disease relapse, 2 from neutropenic sepsis, and 1 from aplasia resulting in hemorrhage and sepsis). Of the remaining 6 patients, 2 relapsed within 1 year post AHCT, 1 relapsed at 3 years post AHCT, and 3 were still being followed at the end of the study period. The overall rate of inadequate hematopoiesis was 67% at 1 year (4 of 6 patients), 33% at 2 years (2 of 6 patients), and 0% (with 1 patient) at 5 years post AHCT.

Multivariate logistic regression showed that pre-treatment with two chemotherapy lines was associated with an increased risk of poor long-term graft function compared to 1 prior chemotherapy line (OR 2.76; 95% CI 1.60–4.78; *p* < 0.001). Other patient and disease characteristics were not independently associated with poor long-term graft function in either univariate or multivariate analysis.

There were 197 PPM patients, 25 of whom were mobilized with preemptive plerixafor and 172 were mobilized with standard regimens. The pre-collection CD34+ count of plerixafor-mobilized versus other PPMs was not significantly different (1.16 × 10^6^ cells/kg versus 1.08 × 10^6^ cells/kg, *p* = 0.480). However, plerixafor-mobilized patients had a significantly higher median CD34+ collection yield when compared to other PPMs (4.048 × 10^6^ cells/kg versus 2.996 × 10^6^ cells/kg, respectively, with *p* = 0.005). All plerixafor-mobilized patients collected >2 × 10^6^ CD34+ cells, whereas 144 of the 197 (74%) PPM patients collected >2 × 10^6^ CD34+ cells/kg. There were no significant differences in the median long-term blood cell counts, rates of poor graft function, transfusion requirements, infection rates, or relapse incidence between plerixafor-mobilized patients and other PPM patients.

In this study, we found that beyond 1 year post-transplant, there was no statistically significant difference in hematopoietic function based on the number of CD34+ cells infused. Previous studies have shown that higher CD34+ doses result in better long-term hematopoietic reconstitution^4,5,8, 9^. Earlier studies that followed patients up to 1 year post-transplant showed that a dose of 3.9 × 10^6^ CD34+ cells/kg was associated with no cytopenias^8^, and >10 × 10^6^ CD34+ cells/kg doses were required to ensure normal peripheral blood counts (WBC >4 × 10^9^/L, hemoglobin >120 g/L, or platelets >150 × 10^9^/L) 6 months post-transplant^4^. These previous studies included patients with non-hematologic malignancies who had undergone multiple lines of treatment, and used higher thresholds for defining normal hematopoietic function, which may account for their findings of increased CD34+ infusion doses required to sustain long-term hematopoietic function.

In our study, patients who were infused <2 × 10^6^ CD34+ cells/kg had a higher incidence of death in the 1 month post-transplant period and only 1 in 10 patients was followed for 5 years post-transplant. Though we found a non-significant trend toward improved hematopoietic function with higher CD34+ doses, given the liberal definition of poor hematopoietic function used in this study, the small differences in the rates of cytopenias did not significantly affect any of the clinical outcomes we looked at. Overall, we found that infusion of <2 × 10^6^ CD34+ cells/kg lead to poor late graft function, and given the lack of statistical or clinically significant improvement in hematopoietic function with doses >3–5 × 10^6^ CD34+, our findings support the transfusion target of 3–5 × 10^6^ CD34+ cells/kg as proposed by the ASBMT. Increasing the target CD34+ above this target would require more apheresis procedures, which comes at an added cost as well as possible risks to the patient (e.g., citrate reactions and thrombocytopenia).

In our study, plerixafor mobilization significantly increased CD34+ collection yield and ensured a collection of >2 × 10^6^ CD34+ cells/kg when compared to standard mobilization regimens for PPM. Prior studies have shown that plerixafor may change the immune composition of the apheresis product, and we hypothesized that this may improve long-term hematopoietic reconstitution^10, 11^. However, similar to our findings, prior studies using plerixafor mobilization have also shown no significant improvement in graft function at 1 year post-transplant^12, 13^. Plerixafor has been shown to increase the quantity of T lymphocytes and natural killer cells in the graft^14, 15^, which may hasten immune recovery and prevent infectious complications. Though our study showed no difference in the infection rate based on mobilization regimen, this may in part be due to low infection rates secondary to the stringent criteria used to define infections (i.e., culture-proven infection or infection requiring hospitalization).

Though subject to the limitations of a retrospective review, this study included a large number of patients and, to our knowledge, reports on the longest follow-up of graft function post AHCT. This study showed that increasing the CD34+ infusion dose >3 × 10^6^ cells/kg did not improve long-term graft function. Also, while preemptive plerixafor increased the collection yield, this did not translate into improved long-term graft function or clinical outcomes. Further studies with larger populations are needed to validate these findings and to determine if increasing CD34+ dose improves the clinical outcomes.
